# Diaphragm ultrasound parameters and rapid shallow breathing index for predicting weaning failure in mechanically ventilated infants: a prospective observational PICU study

**DOI:** 10.1007/s00431-026-07319-1

**Published:** 2026-08-18

**Authors:** Marwa Ibrahem Abdelrazic, Reham Mohamed Mokhtar, Ahmed Hussein Ahmed, Miral Al-Sherif, Abdel-Azeem Mohamed El-Mazary

**Affiliations:** 1 Department of Pediatrics, Faculty of Medicine, Minia University, Minya, Egypt; 2 Department of Pulmonology, Faculty of Medicine, Minia University, Minya, Egypt

**Keywords:** Mechanical ventilation, Weaning failure, Diaphragm ultrasound, Diaphragm thickening fraction, Rapid shallow breathing index, Pediatric intensive care unit, Infants, Spontaneous breathing trial

## Abstract

Weaning from mechanical ventilation in infants admitted to the pediatric intensive care unit remains a challenging clinical decision. Premature extubation may lead to respiratory failure and reintubation, whereas delayed weaning may prolong exposure to invasive ventilation and its complications. Bedside transthoracic ultrasound may provide an objective assessment of diaphragmatic function during spontaneous breathing trials. This study aimed to evaluate the value of diaphragm ultrasound-derived parameters and the rapid shallow breathing index in predicting weaning failure among mechanically ventilated infants in the PICU. This prospective observational study included 40 mechanically ventilated infants aged 28 days to 12 months who were admitted to the PICU of Minia University Children and Maternity Hospital between January 2024 and January 2025 and were considered ready for weaning assessment. Diaphragmatic thickness at inspiration, diaphragmatic thickness at expiration, diaphragm thickening fraction, inferior vena cava diameter, contractility, and rapid shallow breathing index were assessed during the spontaneous breathing trial. The primary outcome was weaning failure, defined as the requirement for post-extubation noninvasive ventilation or reintubation within 24–72 h after extubation. Successful weaning occurred in 34 infants (85.0%) and weaning failure in 6 (15.0%). Infants with weaning failure had significantly lower diaphragmatic thickness at inspiration [1.65 (1.61–1.66 mm) vs. 1.83 (1.79–1.92 mm), *P* < 0.001] and lower diaphragm thickening fraction [12.0 (11.3–13.5%) vs. 26.0 (23.0–28.0%), *P* < 0.001] than successfully weaned infants. RSBI was significantly higher in the weaning failure group [9.0 (9.0–9.8) vs. 7.0 (6.0–7.0), *P* < 0.001]. Diaphragmatic thickness at expiration, inferior vena cava diameter, and contractility did not differ significantly between groups. In this small cohort with only six weaning failure events, apparent ROC AUCs were 0.998 for diaphragmatic thickness at inspiration and 1.000 for both diaphragm thickening fraction and RSBI. After leave-one-out cross-validation of the sample-derived cutoffs, sensitivity/specificity was 83.3%/97.1% for diaphragmatic thickness at inspiration, 83.3%/100.0% for diaphragm thickening fraction, and 100.0%/100.0% for RSBI.

*Conclusion:* In this small single-center cohort with only six weaning failure events, lower diaphragm thickening fraction and diaphragmatic thickness at inspiration, together with higher RSBI, were strongly associated with weaning failure. These findings and the sample-derived cutoffs should be considered exploratory and require external validation in larger multicenter cohorts before routine clinical application.

**Table:** 

**What is Known:** • *Weaning mechanically ventilated infants is challenging, and failed or delayed extubation may increase morbidity.* • *Diaphragm ultrasound and RSBI may support weaning assessment, but infant-specific evidence remains limited.*	
**What is New:** • *Lower inspiratory diaphragm thickness, lower diaphragm thickening fraction, and higher RSBI were associated with weaning failure.* • *Leave-one-out validation retained 100% sensitivity and specificity for RSBI but reduced sensitivity to 83.3% for diaphragmatic thickness at inspiration and diaphragm thickening fraction, underscoring the need for external validation.*

**Supplementary Information:**

The online version contains supplementary material available at https://doi.org/10.1007/s00431-026-07319-1.

## Introduction

Mechanical ventilation is a lifesaving intervention for critically ill infants admitted to the pediatric intensive care unit (PICU). However, prolonged invasive mechanical ventilation is associated with important complications, including ventilator-associated pneumonia, ventilator-induced lung injury, diaphragmatic dysfunction, sedation-related adverse effects, prolonged PICU stay, and increased risk of morbidity. Therefore, timely liberation from mechanical ventilation is a key therapeutic goal in pediatric critical care [1–3].

Weaning from mechanical ventilation is a complex process that requires assessment of respiratory load, respiratory muscle capacity, gas exchange, hemodynamic stability, neurological status, and airway protective reflexes. Premature extubation may lead to respiratory failure, emergency reintubation, and increased complications, whereas delayed extubation may unnecessarily prolong exposure to invasive ventilation and its associated risks [4, 5]. This balance is particularly challenging in infants because respiratory mechanics, chest wall compliance, airway size, and respiratory muscle reserve differ substantially from those of older children and adults.

Traditional readiness parameters, including clinical assessment, spontaneous breathing trials, and indices such as the rapid shallow breathing index (RSBI), are commonly used to support weaning decisions. However, these parameters may not fully reflect diaphragmatic function, which is a central determinant of successful spontaneous breathing after ventilator support is reduced. In mechanically ventilated children, prolonged ventilation and systemic illness may contribute to diaphragmatic weakness, reduced diaphragmatic thickening, and impaired respiratory muscle performance, thereby increasing the risk of weaning failure [1, 6, 7].

Point-of-care ultrasonography has emerged as a noninvasive, bedside, repeatable method for evaluating respiratory muscle function in critically ill patients. Diaphragm ultrasound can assess diaphragmatic thickness during inspiration and expiration, as well as diaphragm thickening fraction, which reflects diaphragmatic contractile activity during respiration. These measurements may provide objective information about diaphragm performance during spontaneous breathing trials and may help identify infants at risk of weaning failure before extubation is attempted [8, 9].

Previous pediatric studies have suggested that diaphragm ultrasound indices, particularly diaphragmatic thickness at inspiration and diaphragm thickening fraction, may help predict weaning or extubation outcomes in mechanically ventilated children [10–12]. Other studies have also evaluated RSBI and combined respiratory indices as predictors of weaning success, with variable performance across different age groups and clinical settings [13, 14]. However, evidence remains limited in mechanically ventilated infants, especially in resource-limited PICU settings where simple bedside tools may be particularly valuable.

Accordingly, the present study aimed to assess the value of transthoracic ultrasound-derived diaphragmatic parameters and RSBI in predicting weaning failure among mechanically ventilated infants admitted to the PICU. Specifically, we evaluated diaphragmatic thickness at inspiration and expiration, diaphragm thickening fraction, RSBI, inferior vena cava diameter, and contractility in relation to weaning outcome. We hypothesized that infants with weaning failure would have lower diaphragm ultrasound indices and higher RSBI compared with infants who were successfully weaned.

## Patients and methods

### Study design and setting

This prospective observational study was conducted in the pediatric intensive care unit (PICU) of Minia University Children and Maternity Hospital, Minia University, Egypt. The study was conducted over 12 months, from January 2024 to January 2025.

The study was designed to evaluate the value of transthoracic ultrasound-derived diaphragmatic parameters and respiratory indices in predicting weaning outcomes among mechanically ventilated infants considered clinically ready for a spontaneous breathing trial.

### Participants

The study included 40 mechanically ventilated infants admitted to the PICU during the study period. Eligible participants were infants aged 28 days to 12 months who required invasive mechanical ventilation and were deemed ready for weaning assessment by the treating PICU team. Age was recorded in months for all participants.

Infants were excluded if they were younger than 28 days of age; had chest wall conditions that could interfere with transthoracic ultrasound assessment, such as rib fractures or chest wall burns; had spinal cord injury; had known congenital lung or pleural malformations; had known neuromuscular disease; were receiving noninvasive ventilatory support only, such as high-flow nasal cannula or Venturi mask; or had hepatosplenomegaly that could interfere with sonographic assessment.

### Ethical considerations

The study protocol was approved by the Medical Ethics Committee of the Faculty of Medicine, Minia University, Egypt (Approval No. 1075/2024). The study was conducted in accordance with the ethical principles of the Declaration of Helsinki and its later amendments and with applicable institutional regulations. Written informed consent was obtained from the parent or legal guardian of each participant before enrollment. Participation was voluntary, and parents or guardians were informed of their right to withdraw from the study at any time without affecting the medical care provided to their child. Patient confidentiality and anonymity were preserved throughout data collection and analysis. The study did not receive any external financial support, and the authors declared no conflict of interest.

### Clinical assessment and data collection

All enrolled infants underwent standardized clinical assessment before the weaning attempt. Baseline data included age, sex, residence, body weight, length, primary diagnosis, duration of mechanical ventilation, and length of PICU stay.

A full clinical history was obtained, including family history, consanguinity, similar previous conditions, presenting symptoms, and duration of ventilatory support. Clinical examination included a general assessment, vital signs, oxygen saturation, nutritional status, skin and lymph node examinations, anthropometric assessment, neurological examination, chest and cardiovascular examinations, and abdominal examination.

Laboratory investigations included complete blood count, C-reactive protein, and arterial blood gas analysis. These measurements were recorded as part of the clinical and physiologic assessment during the weaning evaluation.

### Weaning assessment and outcome definition

All selected infants underwent a spontaneous breathing trial after being judged clinically eligible for weaning by the treating PICU team. The spontaneous breathing trial was performed using pressure support ventilation (PSV) rather than a T-piece. Pressure support and positive end-expiratory pressure levels were determined by the treating PICU team as part of routine clinical care; however, these settings were not protocolized or captured in the study dataset and therefore could not be reported retrospectively. Diaphragmatic ultrasound measurements and RSBI assessment were performed during this PSV-supported trial. Infants were monitored closely for 30 to 120 min during the trial.

The spontaneous breathing trial was considered not tolerated if any of the following occurred: respiratory rate > 45 breaths/min or an increase in respiratory rate > 50% above baseline, arterial oxygen saturation < 90%, arterial oxygen tension < 50 mmHg, increase in arterial carbon dioxide tension > 10 mmHg, increase or decrease in heart rate > 20% from baseline, agitation, diaphoresis, or clinical manifestations of increased work of breathing. If intolerance criteria were developed, the spontaneous breathing trial was terminated, and the infant was returned to the previous mechanical ventilator settings.

The primary outcome was weaning failure. Weaning failure was defined as the requirement for post-extubation noninvasive ventilation or reintubation within 24–72 h after extubation. Successful weaning was defined as successful extubation without either noninvasive ventilation or reintubation during the same post-extubation observation period.

### Transthoracic ultrasound assessment

Transthoracic ultrasound assessment was performed during the spontaneous breathing trial using a Logic ultrasound device. Diaphragmatic ultrasound was performed using a high-frequency linear probe (9–11 MHz). Infants were examined in a semirecumbent position.

The right hemidiaphragm was assessed at the zone of apposition. The linear probe was placed perpendicular to the chest wall in the eighth or ninth intercostal space between the anterior and midaxillary lines. The diaphragm was identified as a three-layered structure composed of two parallel echogenic lines representing the pleura and peritoneum, with a central hypoechoic layer representing the diaphragmatic muscle.

Diaphragmatic thickness was measured at end-inspiration and end-expiration. Measurements were obtained from the central point of the pleural line to the central point of the peritoneal line. Repeated measurements were obtained during each examination, and their average was used for analysis. Only the participant-level averaged values were retained in the analytical dataset; individual replicate measurements and observer-linked paired measurements were unavailable, precluding retrospective estimation of intraobserver or interobserver intraclass correlation coefficients. All ultrasound measurements were completed and recorded during the spontaneous breathing trial before extubation and before the subsequent weaning outcome was determined. Therefore, the operator performing the examination was unaware of the eventual weaning outcome at the time of image acquisition and measurement. However, because the examination was performed at the bedside, the operator was not blinded to the infant’s contemporaneous clinical condition or ventilator support.

The following ultrasound-derived diaphragmatic parameters were recorded:

- Diaphragmatic thickness at inspiration
- Diaphragmatic thickness at expiration
- Diaphragm thickening fraction

Diaphragm thickening fraction was calculated using the following formula:

$$\begin{aligned}\mathrm{DTF}\;(\%)&=[(\text{diaphragmatic thickness at inspiration}-\text{diaphragmatic thickness at expiration})\\ &/\text{diaphragmatic thickness at expiration}]\times 100.\end{aligned}$$

### Rapid shallow breathing index assessment

The rapid shallow breathing index was calculated during the spontaneous breathing trial after stabilization of the breathing pattern. Respiratory rate (breaths/min) and ventilator-displayed tidal volume were recorded contemporaneously. Tidal volume was normalized to body weight and expressed in mL/kg; RSBI was calculated as respiratory rate divided by weight-normalized tidal volume and is therefore reported as breaths/min per mL/kg. Endotracheal tube cuff status, flow-sensor location, and the magnitude of peritubal air leak were not captured in the study dataset; therefore, no standardized leak correction could be applied retrospectively.

### Inferior vena cava and cardiac ultrasound assessment

Inferior vena cava diameter was assessed using a convex array probe in the 2–5 MHz range while the infant was in the supine position. The probe was placed longitudinally on the abdomen in the midline approximately 1 cm below the xiphoid process to visualize the intrahepatic segment of the inferior vena cava. The maximum anterior–posterior inferior vena cava diameter was measured during the expiratory phase of the respiratory cycle.

Cardiac ultrasound assessment was performed as part of hemodynamic evaluation when clinically indicated. Echocardiographic examination was performed in the supine or left lateral decubitus position using a 3S probe. Measurements were obtained according to standard echocardiographic techniques. Left ventricular systolic function was assessed using M-mode measurements, and ejection fraction was calculated using the Teichholz formula.

### Variables

The main predictor variables were diaphragmatic thickness at inspiration, diaphragmatic thickness at expiration, diaphragm thickening fraction, RSBI, inferior vena cava diameter, and contractility percentage.

Additional clinical and laboratory variables included age, body weight, length, sex, primary diagnosis, duration of mechanical ventilation, length of PICU stay, hemoglobin level, total leukocyte count, platelet count, C-reactive protein, arterial pH, PaCO_2_, and bicarbonate level.

The outcome variable was weaning status, classified as successful or failed.

### Study size

A consecutive sampling strategy was used over the fixed recruitment period. Recruitment occurred when mechanically ventilated infants were considered clinically ready for weaning assessment; therefore, the sampling frame comprised infants who reached the spontaneous breathing trial stage rather than all infants who received mechanical ventilation during the study period. All eligible infants who fulfilled the inclusion criteria and underwent weaning assessment were consecutively enrolled. No formal a priori sample size calculation was performed because this was an exploratory, single-center study conducted over a fixed recruitment period. The final analytical sample included 40 infants. A dedicated screening log covering all mechanically ventilated PICU admissions, the number assessed for eligibility, and reason-specific exclusions was not maintained. Consequently, the total number of mechanically ventilated infants screened and the numbers excluded for each reason could not be reconstructed retrospectively.

### Statistical analysis

Data were coded, tabulated, and analyzed using IBM SPSS Statistics version 26.0. Categorical variables were summarized as frequencies and percentages. Continuous variables were assessed for distribution using the Shapiro–Wilk test. Normally distributed variables were presented as mean ± standard deviation, whereas nonnormally distributed variables were presented as median and interquartile range when appropriate.

Comparisons between infants with successful weaning and those with weaning failure were performed using the independent-samples *t* test for normally distributed continuous variables and the Mann–Whitney *U* test for nonnormally distributed continuous variables. Categorical variables were compared using the chi-square test or Fisher’s exact test, as appropriate.

Receiver operating characteristic curve analysis was performed to evaluate the apparent discriminatory performance of ultrasound-derived parameters and RSBI for weaning failure. The area under the curve, 95% confidence interval, optimal cutoff value selected using the Youden index, sensitivity, and specificity were reported for each predictor. Because the cutoffs were derived from a small cohort with only six weaning failure events, their internal stability was assessed using leave-one-out cross-validation (LOOCV). In each of 40 iterations, one infant was omitted, the optimal Youden cutoff was re-estimated using the remaining 39 infants, and the omitted infant was classified using that training-derived cutoff. The 40 held-out classifications were aggregated to calculate cross-validated sensitivity and specificity, with exact binomial 95% confidence intervals. The internal validation was performed using Python version 3.12. Exploratory correlations between the three key predictive parameters—diaphragmatic thickness at inspiration, diaphragm thickening fraction, and RSBI—and 11 clinical or laboratory variables were assessed using Spearman rank correlation coefficients, resulting in 33 pairwise correlation tests. No adjustment for multiple comparisons was applied. Accordingly, the reported correlation *P* values were considered nominal, and associations with *P* < 0.05 were interpreted as hypothesis-generating rather than confirmatory. All statistical tests were two-sided, and a *P* value < 0.05 was considered statistically significant.

## Results

### Study population and baseline clinical characteristics

During the study period, 40 mechanically ventilated infants fulfilled the eligibility criteria and underwent weaning assessment in the PICU. Successful weaning was documented in 34 infants (85.0%), whereas weaning failure occurred in 6 infants (15.0%).

Baseline demographic and clinical characteristics according to weaning outcome are shown in Table 1. The median age of the overall cohort was 6.0 months, and the median body weight was 5.8 kg. Most infants were male (70.0%), and respiratory diagnoses represented the most frequent admission category. There were no statistically significant differences between infants with successful weaning and those with weaning failure regarding age, sex, body weight, length, or respiratory versus nonrespiratory diagnosis.

**Table 1:** Baseline demographic and clinical characteristics according to weaning outcome

Characteristic	Overall cohort (*n* = 40)	Successful weaning (*n* = 34)	Weaning failure (*n* = 6)	*P* value
Age, months	6.0 (3.0–12.0)	6.0 (3.0–12.0)	5.5 (4.2–7.5)	0.985
Male sex	28/40 (70.0)	23/34 (67.6)	5/6 (83.3)	0.648
Body weight, kg	5.8 (4.5–9.0)	5.8 (4.5–9.0)	5.5 (4.6–6.5)	0.569
Length, cm	65.5 (61.0–73.0)	65.5 (61.0–73.0)	65.0 (62.5–66.8)	0.864
Respiratory diagnosis	31/40 (77.5)	26/34 (76.5)	5/6 (83.3)	1.000
PICU stay, days	12.0 (10.0–15.0)	12.0 (10.0–15.0)	14.5 (14.0–15.8)	0.110
Mechanical ventilation duration, days	6.0 (5.0–8.0)	6.0 (5.0–7.0)	9.5 (9.0–10.8)	**0.001**
CRP, mg/L	12.0 (6.0–24.0)	12.0 (6.0–12.0)	48.0 (48.0–84.0)	** < 0.001**

Values are presented as median (interquartile range) for continuous variables and *n*/*N* (%) for categorical variables. Age was recorded in months for all participants. Respiratory diagnosis included pneumonia, bronchiolitis, pneumothorax, bronchial asthma/bronchial airway disease, and other primary respiratory conditions. *P* values for continuous variables were calculated using the Mann–Whitney *U* test because of the small size of the weaning failure group. *P* values for categorical variables were calculated using Fisher’s exact test. Bold values indicate statistical significance at *P* < 0.05

*PICU* pediatric intensive care unit, *CRP* C-reactive protein

Infants with weaning failure had a longer duration of mechanical ventilation than successfully weaned infants [9.5 (9.0–10.8 days) vs. 6.0 (5.0–7.0 days), *P* = 0.001]. PICU stay was also numerically longer in the weaning failure group, but this difference did not reach statistical significance [14.5 (14.0–15.8 days) vs. 12.0 (10.0–15.0 days), *P* = 0.110]. C-reactive protein level was significantly higher among infants with weaning failure than among those with successful weaning [48.0 (48.0–84.0 mg/L) vs. 12.0 (6.0–12.0 mg/L), *P* < 0.001].

### Transthoracic ultrasound and respiratory parameters according to weaning outcome

Transthoracic ultrasound and respiratory parameters according to weaning outcome are shown in Table 2. Infants with weaning failure had significantly lower diaphragmatic thickness at inspiration than successfully weaned infants [1.65 (1.61–1.66 mm) vs. 1.83 (1.79–1.92 mm), *P* < 0.001]. In contrast, diaphragmatic thickness at expiration did not differ significantly between the two groups [1.46 (1.40–1.50 mm) vs. 1.47 (1.42–1.50 mm), *P* = 0.835].

**Table 2:** Transthoracic ultrasound and respiratory parameters according to weaning outcome

Parameter	Overall cohort (*n* = 40)	Successful weaning (*n* = 34)	Weaning failure (*n* = 6)	*P* value
Diaphragmatic thickness at inspiration, mm	1.80 (1.76–1.88)	1.83 (1.79–1.92)	1.65 (1.61–1.66)	** < 0.001**
Diaphragmatic thickness at expiration, mm	1.47 (1.41–1.50)	1.47 (1.42–1.50)	1.46 (1.40–1.50)	0.835
Diaphragm thickening fraction, %	24.5 (22.0–28.0)	26.0 (23.0–28.0)	12.0 (11.3–13.5)	** < 0.001**
Rapid shallow breathing index breaths/min per mL/kg	7.0 (6.0–8.0)	7.0 (6.0–7.0)	9.0 (9.0–9.8)	** < 0.001**
Inferior vena cava diameter, mm	6.7 (5.9–7.7)	6.7 (6.0–7.8)	6.4 (5.7–7.0)	0.609
Contractility, %	65.0 (63.0–66.3)	65.0 (63.3–66.0)	65.5 (60.8–69.5)	0.864

Values are presented as median (interquartile range). *P* values were calculated using the Mann–Whitney *U* test due to the small size of the weaning failure group. Bold values indicate statistical significance at *P* < 0.05

*RSBI* rapid shallow breathing index

Diaphragm thickening fraction was markedly lower among infants with weaning failure than among those with successful weaning [12.0 (11.3–13.5%) vs. 26.0 (23.0–28.0%), *P* < 0.001], indicating reduced diaphragmatic contractile performance during spontaneous breathing. RSBI was significantly higher in the weaning failure group [9.0 (9.0–9.8) vs. 7.0 (6.0–7.0), *P* < 0.001]. Inferior vena cava diameter did not differ significantly between the weaning failure and successful weaning groups [6.4 (5.7–7.0 mm) vs. 6.7 (6.0–7.8 mm), respectively; *P* = 0.609]. Similarly, contractility percentage was comparable between the two groups [65.5 (60.8–69.5%) vs. 65.0 (63.3–66.0%), respectively; *P* = 0.864].

### ROC curve analysis for prediction of weaning failure

Receiver operating characteristic curve analysis was performed to evaluate the apparent discriminatory performance of transthoracic ultrasound-derived parameters and RSBI for weaning failure (Table 3). Diaphragmatic thickness at inspiration showed near-perfect apparent discrimination, with an AUC of 0.998 (95% CI, 0.986–1.000; *P* < 0.001). In the full dataset, a cutoff value of ≤ 1.69 mm achieved 100.0% sensitivity and 97.1% specificity.

**Table 3:** Apparent ROC curve analysis of ultrasound and respiratory parameters for the prediction of weaning failure

Predictor	Direction indicating a higher failure risk	AUC	95% CI	*P* value	Optimal cutoff	Apparent sensitivity	Apparent specificity
Diaphragmatic thickness at inspiration	Lower values	0.998	0.986–1.000	** < 0.001**	≤ 1.69 mm	100.0%	97.1%
Diaphragm thickening fraction	Lower values	1.000	1.000–1.000	** < 0.001**	≤ 18.0%	100.0%	100.0%
Rapid shallow breathing index, breaths/min per mL/kg	Higher values	1.000	1.000–1.000	** < 0.001**	≥ 9.0	100.0%	100.0%
Diaphragmatic thickness at expiration	Lower values	0.529	0.258–0.795	0.835	≤ 1.42 mm	50.0%	73.5%
Inferior vena cava diameter	Lower values	0.569	0.236–0.892	0.609	≤ 5.8 mm	50.0%	82.4%
Contractility	Lower values	0.475	0.068–0.884	0.864	≤ 63.0%	50.0%	73.5%

The outcome variable was weaning failure. Cutoff values were selected using the Youden index. The sensitivity and specificity reported in the table are apparent estimates obtained using cutoffs derived from the full dataset; leave-one-out cross-validation estimates are reported in “Results.” For diaphragmatic thickness at inspiration, diaphragmatic thickness at expiration, diaphragm thickening fraction, inferior vena cava diameter, and contractility, lower values were evaluated as indicating a higher risk of weaning failure. For RSBI, higher values were evaluated as indicating a higher risk of weaning failure. *P* values reflect between-group differences and should be interpreted with caution because the weaning failure group included only 6 infants. Bold values indicate statistical significance at *P* < 0.05

*ROC* receiver operating characteristic, *AUC* area under the curve, *CI* confidence interval, *RSBI* rapid shallow breathing index

Diaphragm thickening fraction also showed perfect apparent discrimination, with an AUC of 1.000 (95% CI, 1.000–1.000; *P* < 0.001). In the full dataset, a cutoff value of ≤ 18.0% achieved 100.0% sensitivity and 100.0% specificity for identifying infants with weaning failure. RSBI similarly demonstrated perfect apparent discrimination, with an AUC of 1.000 (95% CI, 1.000–1.000; *P* < 0.001). In the full dataset, a cutoff value of ≥ 9.0 breaths/min per mL/kg achieved 100.0% sensitivity and 100.0% specificity.

In contrast, diaphragmatic thickness at expiration, inferior vena cava diameter, and contractility percentage showed poor discriminatory performance for predicting weaning failure. These findings suggest that inspiration-related diaphragmatic thickening parameters and RSBI were the most informative measures for predicting weaning failure in this cohort. In contrast, static expiratory thickness, IVC diameter, and contractility were less useful.

Internal validation showed differing cutoff stability across the three leading predictors. During leave-one-out cross-validation, the training-derived cutoff for diaphragmatic thickness at inspiration ranged from ≤ 1.66 to ≤ 1.69 mm; aggregate held-out sensitivity was 83.3% (5/6; exact 95% CI, 35.9–99.6) and specificity was 97.1% (33/34; exact 95% CI, 84.7–99.9). For diaphragm thickening fraction, training-derived cutoffs ranged from ≤ 14.0 to ≤ 18.0%, with sensitivity of 83.3% (5/6; exact 95% CI, 35.9–99.6) and specificity of 100.0% (34/34; exact 95% CI, 89.7–100.0). The RSBI cutoff remained ≥ 9.0 in all iterations and yielded sensitivity of 100.0% (6/6; exact 95% CI, 54.1–100.0) and specificity of 100.0% (34/34; exact 95% CI, 89.7–100.0). Thus, the RSBI cutoff was unchanged across the leave-one-out iterations, whereas the exact cutoffs for diaphragmatic thickness at inspiration and diaphragm thickening fraction were influenced by individual observations.

### Correlation analysis of key predictive parameters

Exploratory Spearman correlation analysis between the three key predictive parameters and 11 clinical or laboratory variables is presented in Table S1. Of the 33 pairwise correlations examined, 12 had nominal *P* values < 0.05; no adjustment for multiple comparisons was applied. Diaphragmatic thickness at inspiration was positively correlated with age (*ρ* = 0.41, *P* = 0.009), body weight (*ρ* = 0.58, *P* < 0.001), and platelet count (*ρ* = 0.41, *P* = 0.008) and negatively correlated with PICU stay (*ρ* = − 0.40, *P* = 0.011), mechanical ventilation duration (*ρ* = − 0.44, *P* = 0.005), and total leukocyte count (*ρ* = − 0.35, *P* = 0.026). Diaphragm thickening fraction was positively correlated with platelet count (*ρ* = 0.33, *P* = 0.037) and negatively correlated with C-reactive protein (*ρ* = − 0.34, *P* = 0.044). RSBI was positively correlated with mechanical ventilation duration (*ρ* = 0.37, *P* = 0.018) and C-reactive protein (*ρ* = 0.42, *P* = 0.012) and negatively correlated with platelet count (*ρ* = − 0.48, *P* = 0.002) and arterial pH (*ρ* = − 0.37, *P* = 0.020). Given the number of comparisons and the cohort size, these unadjusted associations may include chance findings and should be interpreted solely as hypothesis-generating.

### Visual summary of key findings

**Fig. 1:**
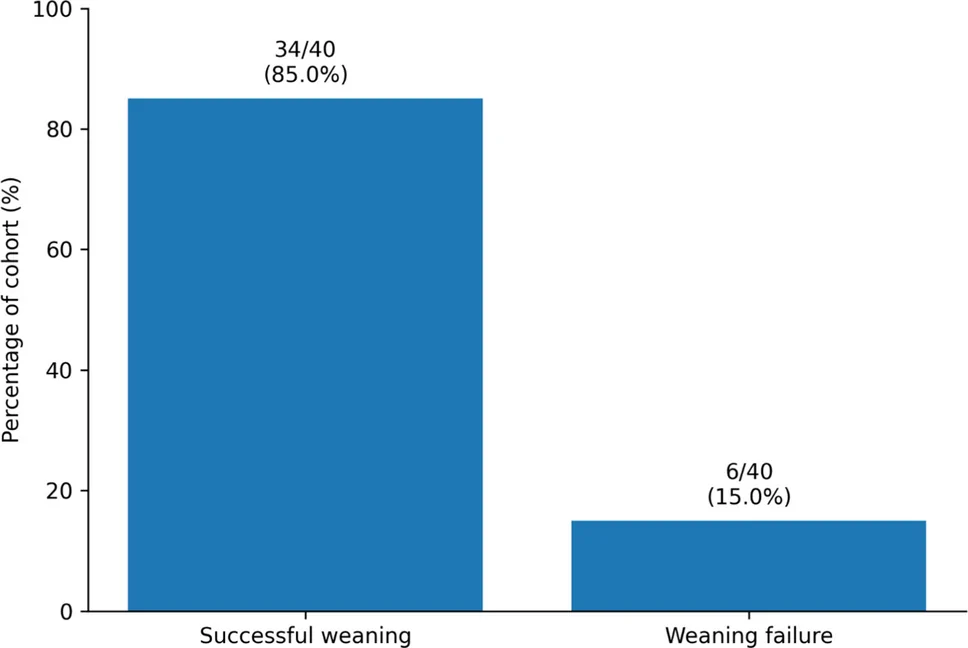
Distribution of weaning outcomes in mechanically ventilated infants. The figure shows the distribution of weaning outcomes in the analytical cohort. Successful weaning occurred in 34 of 40 infants (85.0%), whereas weaning failure occurred in 6 of 40 infants (15.0%)

**Fig. 2:**
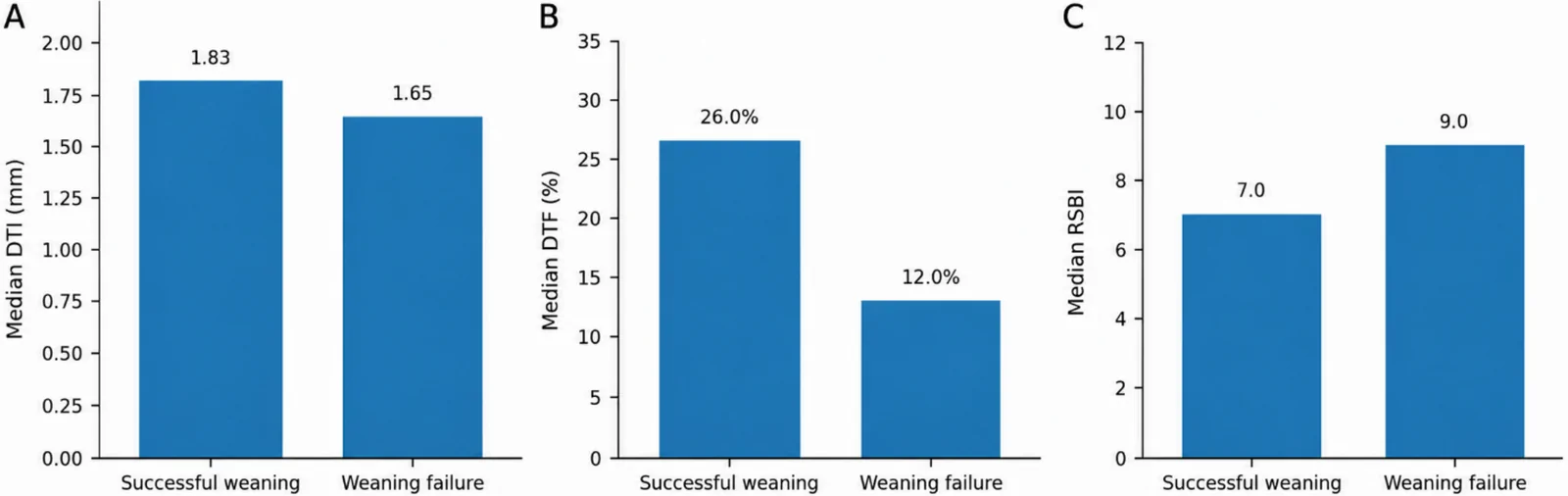
Diaphragm ultrasound-derived parameters and rapid shallow breathing index according to weaning outcome. **A** Median diaphragmatic thickness at inspiration (DTI) was lower in infants with weaning failure than in those with successful weaning. **B** Median diaphragm thickening fraction (DTF) was markedly lower in the weaning failure group, indicating reduced diaphragmatic contractile performance during the spontaneous breathing trial. **C** Median rapid shallow breathing index (RSBI) was higher in infants with weaning failure, reflecting a more rapid and shallow breathing pattern during weaning assessment. Between-group differences for DTI, DTF, and RSBI were statistically significant (all *P* < 0.001). Values shown above bars represent group medians

**Fig. 3:**
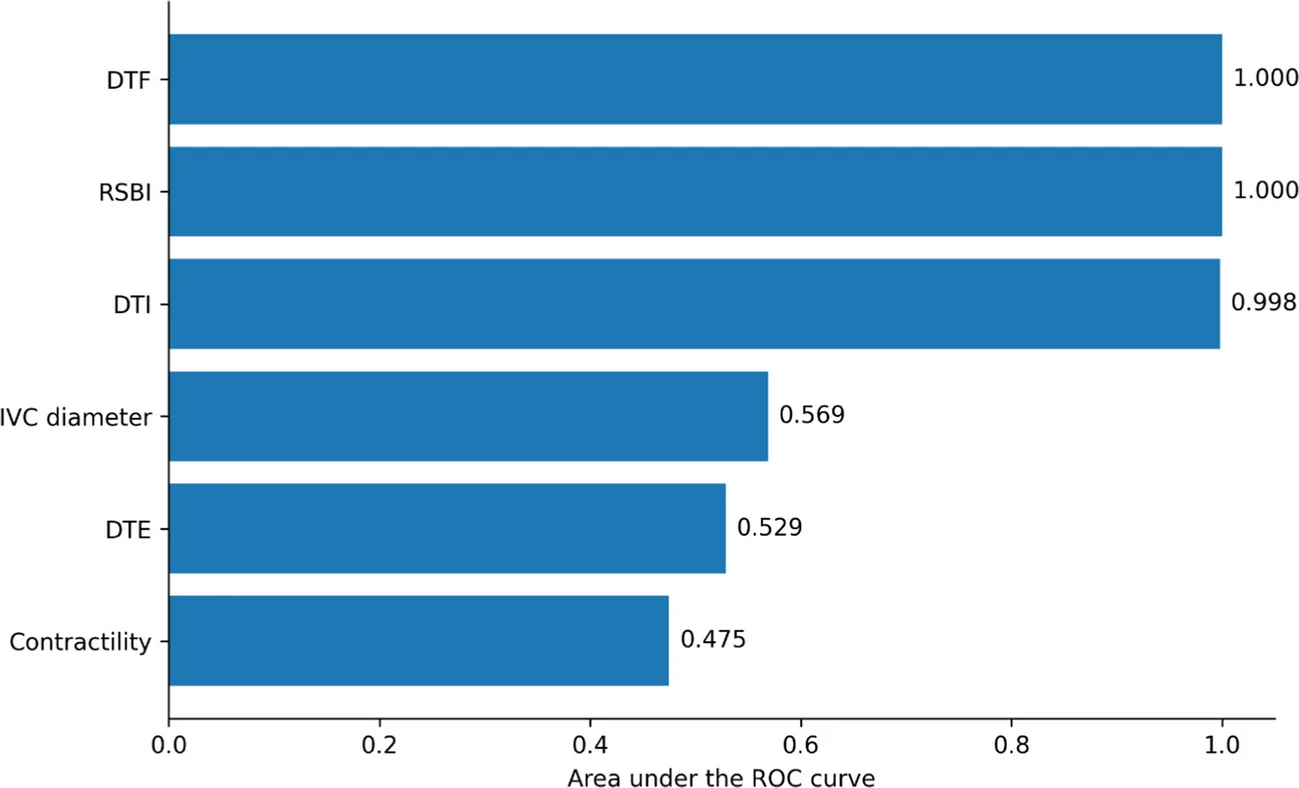
ROC-derived predictive performance of ultrasound and respiratory parameters for weaning failure. The figure summarizes the apparent area under the receiver operating characteristic curve for each evaluated parameter using the full dataset. Diaphragm thickening fraction, RSBI, and diaphragmatic thickness at inspiration showed the highest apparent discriminatory performance. In contrast, diaphragmatic thickness at expiration, inferior vena cava diameter, and contractility showed poor apparent discriminatory performance. Internal validation of the sample-derived cutoffs is reported separately in “Results.” *ROC* receiver operating characteristic, *DTI* diaphragmatic thickness at inspiration, *DTE* diaphragmatic thickness at expiration, *DTF* diaphragm thickening fraction, *RSBI* rapid shallow breathing index, *IVC* inferior vena cava. ROC findings should be interpreted with caution because the weaning failure group comprised only six infants

A visual summary of the principal findings is presented in Figs. 1, 2, and 3. Figure 1 shows the distribution of weaning outcomes in the study cohort. Figure 2A–C compares the main predictive parameters by weaning outcome, highlighting lower diaphragmatic thickness at inspiration, a lower diaphragm thickening fraction, and a higher RSBI among infants with weaning failure. Figure 3 summarizes the ROC-derived discriminatory performance of the evaluated ultrasound and respiratory parameters for predicting weaning failure.

## Discussion

In this prospective observational study of mechanically ventilated infants admitted to the PICU, diaphragmatic ultrasound parameters and RSBI were closely associated with weaning outcome. Infants who experienced weaning failure had significantly lower diaphragmatic thickness at inspiration, markedly lower diaphragm thickening fraction, and higher RSBI compared with successfully weaned infants. In contrast, diaphragmatic thickness at expiration, inferior vena cava diameter, and contractility percentage did not differ significantly between outcome groups. ROC analysis showed very high apparent discriminatory performance for diaphragmatic thickness at inspiration, diaphragm thickening fraction, and RSBI, whereas inferior vena cava diameter and contractility showed poor discriminatory value. These findings suggest that dynamic measures of diaphragmatic recruitment and respiratory load during spontaneous breathing may be more clinically informative than static or volume-based measurements for assessing weaning readiness in mechanically ventilated infants.

The process of weaning children from invasive mechanical ventilation is complex. It requires the integration of respiratory mechanics, respiratory muscle capacity, gas exchange, hemodynamic stability, neurological status, and airway-protective reflexes. Both premature and delayed extubation carry important risks. Premature extubation may lead to respiratory failure, emergency reintubation, and increased morbidity. In contrast, unnecessary prolongation of invasive ventilation may increase the risk of ventilator-associated complications, sedation exposure, diaphragmatic dysfunction, and prolonged PICU stay [1, 3, 4]. In infants, this decision is particularly challenging because of developmental differences in airway size, chest wall compliance, respiratory muscle reserve, and vulnerability to fatigue. Therefore, objective bedside tools that can complement clinical judgment may be especially valuable in this population.

The most important finding in the present study was the strong association between reduced diaphragm thickening fraction and weaning failure. Diaphragm thickening fraction reflects the relative increase in diaphragmatic thickness during inspiration and is commonly interpreted as a surrogate marker of diaphragmatic contractile activity. A low DTF during a spontaneous breathing trial suggests impaired diaphragmatic recruitment and reduced ability to sustain spontaneous ventilation after ventilatory support is withdrawn. This finding is consistent with previous pediatric studies reporting that lower DTF is associated with extubation or weaning failure in mechanically ventilated children [10–12]. The present results therefore support the use of diaphragm ultrasound as a practical bedside method for identifying infants at increased risk of weaning failure.

Diaphragmatic thickness at inspiration was also significantly lower among infants with weaning failure. Unlike expiratory thickness, inspiratory thickness reflects the ability of the diaphragm to thicken during active contraction. The lack of a significant difference in diaphragmatic thickness at expiration in this study suggests that static diaphragm thickness alone may be less informative than dynamic inspiratory thickening. This distinction is clinically important. An infant may have preserved baseline diaphragm thickness but still fail to generate sufficient inspiratory thickening during spontaneous breathing. Therefore, inspiratory thickness and thickening fraction may better reflect functional readiness for liberation from mechanical ventilation than resting expiratory thickness.

RSBI was significantly higher among infants with weaning failure and showed excellent apparent discrimination in ROC analysis. A higher RSBI reflects a breathing pattern characterized by rapid shallow respiration, which may indicate increased respiratory load, reduced tidal volume generation, respiratory muscle fatigue, or poor reserve during spontaneous breathing. Although RSBI has been widely studied in adults, its performance in pediatric populations is more variable because respiratory rate and tidal volume change substantially with age and body size. Previous pediatric and mixed critical care studies have reported variable predictive performance for RSBI, with some suggesting useful predictive value and others showing that ultrasound-derived diaphragmatic indices may perform better [12–14]. In the current cohort, RSBI performed well, but this should be interpreted with caution given the small number of weaning failure events. Furthermore, endotracheal tube cuff status and peritubal leak magnitude were not systematically recorded; consequently, measurement error in ventilator-derived tidal volume may have affected the calculated RSBI and its apparent predictive performance.

The ventilatory conditions during measurement should also be considered when comparing the present findings with previous pediatric studies. All DTF and RSBI measurements in this study were obtained during a PSV-supported spontaneous breathing trial rather than during a T-piece trial. Pressure support partially unloads the inspiratory muscles and may underestimate the breathing effort required after extubation in children [15]. It may consequently reduce diaphragmatic activation and DTF while increasing tidal volume and/or reducing respiratory rate, thereby tending to lower RSBI relative to measurements obtained during a less-assisted or T-piece trial. Therefore, the absolute DTF and RSBI values and their sample-derived cutoffs should be considered specific to the ventilatory conditions under which they were measured and should not be regarded as directly interchangeable with values obtained using T-piece, CPAP-only, or different pressure-support settings. Because the exact pressure support and PEEP levels were neither standardized nor recorded, the magnitude of this effect could not be quantified in the present cohort.

Inferior vena cava diameter did not differ significantly between infants with successful weaning and those with weaning failure and showed poor discriminatory performance in ROC analysis. This suggests that IVC diameter, as measured in this cohort, was not a useful standalone predictor of weaning outcome. Although fluid status and cardiopulmonary interaction may influence weaning tolerance, a single IVC diameter measurement may not adequately capture dynamic preload responsiveness, pulmonary congestion, cardiac function, or respiratory effort in mechanically ventilated infants. Similarly, the contractility percentage was not significantly associated with weaning outcome and showed no meaningful predictive performance. These findings support prioritizing diaphragm-specific ultrasound parameters over less specific hemodynamic or static measurements when the clinical question is readiness for ventilator liberation.

The correlation analysis provides additional physiologic context. Diaphragmatic thickness at inspiration was positively correlated with age and body weight, suggesting that older and heavier infants tended to have greater diaphragmatic thickness. It was also negatively correlated with the duration of mechanical ventilation and PICU stay, consistent with the concept that prolonged critical illness and ventilatory support may contribute to respiratory muscle weakness or reduced diaphragmatic performance. RSBI was positively correlated with mechanical ventilation duration and C-reactive protein and negatively correlated with arterial pH and platelet count, suggesting that greater inflammatory burden and physiologic derangement may be associated with a less efficient breathing pattern during weaning assessment. However, 33 pairwise correlations were examined without adjustment for multiple comparisons in a cohort of 40 infants. The reported *P* values are therefore nominal, and associations close to the 0.05 threshold may represent chance findings. These correlations should not be interpreted causally or as independently confirmed relationships; rather, they provide preliminary, hypothesis-generating observations that require replication in larger cohorts.

The ROC results are clinically promising but require cautious interpretation. DTF and RSBI showed perfect apparent discrimination in this dataset, and DTI showed near-perfect apparent discrimination. Internal validation revealed a more nuanced pattern: Leave-one-out sensitivity decreased to 83.3% for both DTI and DTF, although specificity remained 97.1% and 100.0%, respectively, whereas RSBI retained 100.0% sensitivity and specificity. This distinction is important because the rank separation in the full cohort was striking, but the exact DTI and DTF thresholds changed when individual observations were omitted. Moreover, because only six infants experienced weaning failure, the cross-validated estimates remained imprecise, as reflected by their wide exact confidence intervals. The proposed thresholds should therefore be considered exploratory and hypothesis-generating rather than definitive clinical decision thresholds. External validation in a larger multicenter cohort remains necessary before these cutoffs can be recommended for routine clinical use.

This study has several strengths. It focused on a clinically important PICU decision point: whether a mechanically ventilated infant is ready for weaning and extubation. The study also evaluated bedside, noninvasive, repeatable measurements that are feasible in routine critical care practice. Another strength is the simultaneous assessment of diaphragm ultrasound parameters, RSBI, IVC diameter, contractility, and clinical/laboratory variables, allowing a broader evaluation of respiratory and physiologic factors related to weaning outcome. The use of ROC analysis and correlation analysis also provides clinically interpretable information about both discriminatory performance and physiologic associations.

Several limitations should be acknowledged. First, this was a single-center study with a small sample size determined by a fixed recruitment period rather than by a formal a priori sample size calculation. The particularly small weaning failure group limits statistical power and increases the risk of unstable estimates, especially for ROC-derived cutoffs; internal validation cannot fully compensate for the small number of outcome events. Second, recruitment occurred when infants were judged clinically ready for weaning assessment, and a dedicated screening log covering all mechanically ventilated PICU admissions was not maintained. Therefore, the total number screened and the numbers excluded for each reason could not be reported. Although eligible infants were recruited consecutively, the representativeness of the final cohort and the potential for selection bias cannot be fully assessed. Third, ultrasound measurements may be operator-dependent. Although repeated measurements were averaged for analysis, individual replicate measurements and independent second-operator measurements were not retained. Consequently, intraobserver and interobserver reliability could not be evaluated using intraclass correlation coefficients. Fourth, the spontaneous breathing trial was performed using PSV rather than a T-piece, and the exact pressure support and PEEP levels were neither protocolized nor prospectively recorded. Pressure support may unload the inspiratory muscles and alter respiratory rate, tidal volume, DTF, and RSBI. This limits direct comparison with studies that used a T-piece, CPAP-only, or different ventilatory support levels and may have influenced the study-derived thresholds. Fifth, RSBI was calculated using ventilator-displayed tidal volume normalized to body weight. However, endotracheal tube cuff status, flow-sensor location, and the magnitude of peritubal air leak were not prospectively captured, and no standardized leak correction was applied. An air leak, particularly with an uncuffed or inadequately sealed endotracheal tube, may introduce error into the ventilator-displayed tidal volume and, consequently, into the calculated RSBI. This measurement uncertainty may have influenced the apparently strong discriminatory performance of RSBI. Sixth, the study included infants only, so the findings may not be directly generalizable to older children or mixed pediatric populations. Seventh, although the study was prospective, it was observational; therefore, associations between ultrasound parameters and weaning outcome should not be interpreted as causal. Eighth, in this study, weaning failure was defined as the requirement for post-extubation noninvasive ventilation or reintubation within 24–72 h after extubation. Because local clinical thresholds and PICU practices may influence initiation of noninvasive ventilation, the observed failure rate may not be directly comparable with studies that define failure using reintubation alone.

In conclusion, this study suggests that diaphragm ultrasound-derived measures, particularly diaphragm thickening fraction and diaphragmatic thickness at inspiration, together with RSBI, may provide useful bedside information for predicting weaning failure in mechanically ventilated infants. Static expiratory thickness, IVC diameter, and contractility appeared less informative in this cohort. Because the number of weaning failure events was small, the proposed cutoff values should be interpreted as exploratory. Larger multicenter studies with standardized ultrasound protocols, assessment of interobserver reliability, and external validation of cutoff values are needed before these measures can be incorporated into routine pediatric weaning algorithms.

## Conclusion

In this prospective observational PICU study, diaphragm ultrasound-derived parameters and RSBI were associated with weaning outcome in mechanically ventilated infants. Infants with weaning failure had significantly lower diaphragmatic thickness at inspiration, markedly lower diaphragm thickening fraction, and higher RSBI compared with successfully weaned infants. In contrast, diaphragmatic thickness at expiration, inferior vena cava diameter, and contractility showed limited predictive value.

In this small single-center cohort with only six weaning failure events, diaphragm thickening fraction, diaphragmatic thickness at inspiration, and RSBI demonstrated excellent apparent discriminatory performance for identifying infants at risk of weaning failure. Leave-one-out cross-validation retained 100.0% sensitivity and specificity for RSBI. Still, it reduced sensitivity to 83.3% for diaphragmatic thickness at inspiration and the diaphragm thickening fraction, indicating that individual observations influenced some of the sample-derived cutoffs. These findings and proposed cutoff values should therefore be interpreted as exploratory and hypothesis-generating. Larger multicenter studies with standardized ultrasound and spontaneous breathing trial protocols, assessment of interobserver reliability, and external validation are needed before these parameters can be incorporated into routine pediatric weaning algorithms.

## Supplementary Information

Below is the link to the electronic supplementary material.

ESM 1 (DOCX 15.6 KB)

## Data Availability

No datasets were generated or analysed during the current study.
