# Supplementary material for: Diaphragm ultrasound parameters and rapid shallow breathing index for predicting weaning failure in mechanically ventilated infants: a prospective observational PICU study
Source: Eur J Pediatr. 2026 Aug 18;185(9):675. doi: 10.1007/s00431-026-07319-1 (PMC13486014; doi:10.1007/s00431-026-07319-1)
Supplement: Supplementary file 1 [file 431_2026_7319_MOESM1_ESM.docx]

# Table S1. Correlations of key predictive parameters with clinical and laboratory variables

| Variable | DTI, ρ (P value) | DTF, ρ (P value) | RSBI, ρ (P value) |
| --- | --- | --- | --- |
| Age, months | **0.41 (0.009)** | 0.04 (0.788) | −0.13 (0.441) |
| Body weight, kg | **0.58 (<0.001)** | 0.05 (0.740) | −0.05 (0.743) |
| PICU stay, days | **−0.40 (0.011)** | −0.20 (0.213) | 0.30 (0.061) |
| Mechanical ventilation duration, days | **−0.44 (0.005)** | −0.28 (0.085) | **0.37 (0.018)** |
| Hemoglobin, g/dL | −0.12 (0.458) | 0.09 (0.579) | −0.11 (0.518) |
| Total leukocyte count, ×10⁹/L | **−0.35 (0.026)** | −0.28 (0.076) | 0.31 (0.055) |
| Platelet count, ×10⁹/L | **0.41 (0.008)** | **0.33 (0.037)** | **−0.48 (0.002)** |
| C-reactive protein, mg/L | −0.25 (0.144) | **−0.34 (0.044)** | **0.42 (0.012)** |
| Arterial pH | 0.17 (0.306) | 0.12 (0.468) | **−0.37 (0.020)** |
| PaCO₂, mmHg | −0.05 (0.776) | −0.12 (0.461) | −0.09 (0.576) |
| HCO₃⁻, mmol/L | −0.23 (0.148) | −0.12 (0.452) | 0.17 (0.297) |

Values are Spearman rank correlation coefficients (ρ) with corresponding two-sided P values. DTI, diaphragmatic thickness at inspiration; DTF, diaphragm thickening fraction; RSBI, rapid shallow breathing index; PICU, pediatric intensive care unit; PaCO₂, partial pressure of arterial carbon dioxide; HCO₃⁻, bicarbonate. A total of 33 pairwise correlations were examined, and no adjustment for multiple comparisons was applied. Accordingly, all reported P values are nominal, and associations with P < 0.05 should be regarded as exploratory and hypothesis-generating rather than confirmatory or causal. C-reactive protein correlations were based on available data. Bold values indicate nominal P < 0.05 and do not represent multiplicity-adjusted statistical significance.
